# Prevalence and Assessment of Factors Associated With Malnutrition in Children Residing in Slums of Mumbai: A Cross-Sectional Study

**DOI:** 10.7759/cureus.58619

**Published:** 2024-04-19

**Authors:** Pratyush Kumar, Kumar Abhishek, Rushikesh Shukla, Manali Sarkar, GP Kaushal, Pankaj Gharde, Urmil Shah, Suhrud Panchawagh, Shabarini Srikumar

**Affiliations:** 1 Department of Medicine, Dr Baba Saheb Ambedkar Medical College and Hospital, Delhi, IND; 2 Department of Medicine, Jawaharlal Nehru Medical College, Datta Meghe Institute of Higher Education and Research, Wardha, IND; 3 Department of Internal Medicine, MGM Medical College, Navi Mumbai, IND; 4 Department of Pediatrics, Dr Baba Saheb Ambedkar Medical College and Hospital, Delhi, IND; 5 Department of General Surgery, Jawaharlal Nehru Medical College, Datta Meghe Institute of Higher Education and Research, Wardha, IND; 6 Department of Medicine, Rajiv Gandhi Medical College and Chhatrapati Shivaji Maharaj Hospital, Mumbai, IND; 7 Department of Medicine, Smt. Kashibai Navale Medical College and General Hospital, Pune, IND; 8 Department of Internal Medicine, Tirunelveli Medical College, Tirunelveli, IND

**Keywords:** mumbai, india, slums, child health, malnutrition, under 5 age group

## Abstract

Background

Malnutrition in children continues to be a serious public health problem in India. Therefore, this study aims to evaluate the prevalence of malnutrition and assess factors contributing to it in children of the marginalized slum population of India, masked in the metropolitan cities.

Methods

A retrospective data analysis with a cross-sectional model was conducted by medical volunteers affiliated with the Rotaract Club of Medicrew who had organized a free pediatric health check-up camp in the Dharavi village of Mumbai, India for children under five. Children under five years of age group of either sex residing in the slums of Dharavi and whose parents consented are included in the study. Neonates, children older than five years of age, and children whose parents did not consent for them to be included in the study were excluded. A pretested, pre-validated questionnaire was administered, and statistical analysis was done with p-values <0.05 considered to be statistically significant.

Results

A total of 126 children were included. Out of these children, 109 of them (86.50%) had a mid-arm circumference of more than 12.5 cm (normal), 11 (8.73%) were between 11.5 cm and 12.5 cm (moderate acute malnutrition), and five (4.77%) were less than 11.5 cm (severe acute malnutrition). Among the 126 kids, 86 kids were above the age of two and their BMI was assessed, 36 (44.19%) were found to be underweight (<5th percentile) while 14 (16.3%) were obese (>95th percentile), and four (4.65%) were overweight (85th-95th percentile). For 106 (84.13%) of these children, the caregivers were mothers while others were fathers (n=4; 3.18%), grandmothers (n=5; 3.97%), sisters (n=5; 3.97%), and aunts (n=6; 4.76%). Out of those who had commenced receiving formal education, only 39 (55.71%) were in an appropriate grade for their age. The mean expenditure on food as a proportion of the total household income was 36.40% (standard deviation (SD) 15.0%). On the single-item sleep quality scale, the sleep of only 36 kids (28.58%) was reported by their caregivers as excellent. A high proportion of other medical problems were reported in the children.

Conclusion

Our study reports a substantial burden of malnutrition among children residing in the slums of Dharavi. Rigorous strengthening and conceptualization of on-ground nutritional programs targeted toward slum children should be done by Indian healthcare policymakers.

## Introduction

Malnutrition is defined as the imbalance (either excess or inadequate) intake of nutrients and calories in an individual [1]. The inadequate intake of nutrients is referred to as undernutrition and is further subcategorized into stunting, wasting, and underweight. Before elaborating on the definition of each of the subcategories of undernutrition, it is important to understand what is a Z-score. The Z-score is the difference between the observed value and the median value divided by the standard deviation (SD). In the sphere of nutrition, it is calculated as the difference between the observed and the age- and sex-appropriate median value of the standard population divided by the SD of the standard population. Current nutritional studies refer to the standard population from those noted by the WHO growth standards with this background in mind; wasting is defined as a weight-for-height Z-score (WHZ) <2 SD. Similarly, underweight is defined as a weight-for-age Z-score <2 SD, and stunting is defined as a height-for-age Z-score <2 SD. Undernutrition can also be subcategorized based on onset into acute and chronic malnutrition. Weight-for-height is the best marker for acute onset malnutrition whereas height-for-age is found to be a phenomenal marker for chronic malnutrition. The indicator that is most frequently used is weight-for-age. The excess intake of nutrients leads to a paradoxical condition called obesity where although the energy consumption exceeds the daily requirements, there is a shortage of essential micronutrients.

Malnutrition in children continues to be a serious public health problem in India. India has one of the highest rates of malnourished children among developing nations. In India, about half of all children are malnourished, and every year, nearly a million children die before they turn one month old [1]. Despite being a major contributor to the global economy and especially for a country going through a developmental drift, the country’s growth rates are far greater than the 20% threshold that needs to be achieved. The existing Global Nutrition Targets for 2025 and the Sustainable Development Goals 2030: goal 2 seem far from being achieved from their target years [2].

This is also prevalent in metropolitan cities of India where the increasing migrating population and lack of resources have divided lines between the urban and sub-urban slum areas. The United Nations has defined slum living as having insufficient access to safe water, sanitation, basic services, adequate living space, durable housing, and protection from forcible eviction [2]. Slums are home to one billion people worldwide, including roughly half of Mumbai's inhabitants in 2011 [2]. These slums act as concentration camps for the marginalized population of the metropolitan cities who have been driven out of their villages in search of better livelihood. Children are the most vulnerable population in these slums exposed to repetitive infections, poor livelihood conditions, and poor dietary intake predisposing them to malnutrition.

In a study conducted in 2018 by Hemalatha et al., urban slums had greater rates of stunting, underweight, and anemia among early children, but overall Mumbai had lower rates of wasting [3]. Hemalatha et al. also reported that compared to non-slum areas, children in urban slums experience a higher proportion of undernutrition [3]. Additionally, gender differences in health and nutrition status have also been found in numerous studies [3]. Currently, India is tackling this problem via targeted nutritional programs like Integrated Child Development Services (ICDS) applicable universally. Therefore, no additional or specialized focus is given to these hubs of malnutrition at the moment, and this study aims to highlight the importance of introducing a region-wise (based on socio-economics) approach to alleviation of the situation.

Understanding the need of the hour, growth monitoring and surveillance are considered to play a pivotal role in child health programs [3]. Malnutrition may be then divided into two types based on the onset, being chronic and acute malnutrition. Weight-for-height is the best marker for acute onset malnutrition whereas height-for-age is found to be a phenomenal marker for chronic malnutrition [1]. The indicator that is most frequently used is weight-for-age [1].

As per WHO criteria, global acute malnutrition (GAM) or wasting is defined as children with WHZ <2 SD and/or mid-upper arm circumference (MUAC) <125 mm and/or the presence of bilateral pitting edema [4]. Stunting, as determined by height-for-age, and underweight, as determined by a decrease in weight-for-age, regularly occur and are correlated, particularly so in the Indian context [5]. Underweight, stunting, and wasting all had prevalence rates of 35.7%, 33.8%, and 18.5%, respectively, all across the country. Severe wasting or acute malnutrition are signs of low MUAC (11.5 cm), which also indicates sickness and a risk of mortality [5]. Stunting incidence varies from 20% in Goa and Kerala, which score relatively well on most health metrics, to 48% in Bihar, with an average prevalence of 38.4% across India [3]. In comparison to the majority of the southern and northeastern states, where stunting incidence is low (30%), all of the central and eastern states as well as the majority of the western states have a significant burden of stunting (>30%) [3].

The burden is evenly distributed between the northern states. This might be a result of the cultural and nutritional customs prevalent in the northeastern states, where flesh-eating is more prevalent [3]. In Mumbai's slums compared to non-slum areas, stunting, and underweight rates were both 40% and 14% higher, respectively [3].

There are various modes of intervention that can be implemented to prevent malnutrition. Interventions like supplementary feeding programs, education related to nutrition, food enrichment, fortification of biscuits, and novel services like family planning.

Therefore, this study aims to evaluate the prevalence of malnutrition and assess factors contributing to it in children under the age of five group residing in the marginalized slum population of Mumbai, India masked in the crowd as a metropolitan city.

## Materials and methods

Study design

This is a retrospective data analysis with a cross-sectional model.

Study population

Children under five years of age who were beneficiaries of the health check-up camps conducted by the Rotaract Club of Medicrew in the Dharavi slum area of Mumbai over a period of two months.

Inclusion criteria

Children under five years of age of either sex residing in the slums of Dharavi, Mumbai, and whose parents consented for them to be beneficiaries of the health camp are included in the study.

Exclusion criteria

Neonates and children older than five years of age with any history of diagnosed chronic medical conditions or syndromes were excluded from the study. Children who were visiting the area and not residents of the slums were also excluded during data analysis.

Consent

An oral well-informed consent explaining to the parents of the children about the methodology and implications of the screening camp and derived study was taken. All participants' details are confidential and anonymized. Ethical clearance was taken from the Institutional Ethical Clearance Committee of Datta Meghe Institute of Medical Sciences (DMIMS(DU)/IEC/2022/285).

Study tool

A pretested, pre-validated questionnaire was administered to the consenting participants of the study. The questionnaire had eight sections, which were designed in consultations with pediatricians; they were as follows: 1. child details; 2. caregiver details; 3. child medical and social history; 4. child general examination; 5. child gastrointestinal (GI) history; 6. Child Eating Behavior Questionnaire (desire to drink, satiety responsiveness, and food responsiveness) 7. child weight history; 8. cognitive function tests (attitude, behavior, speech, thought, movements, and cognition). The question is available upon request. 

Methodology 

After obtaining the informed consent of the caregiver, the above-mentioned study tool was administered to the participant via a healthcare volunteer who was part of a health check-up camp from the Rotaract Club of Medicrew. The volunteers noted down all the details and information as per the prescribed study tool in a predesigned Google form. The data was collected from four different lanes chosen randomly in the Dharavi slum of Mumbai. This data was retrospectively investigated. 

Sample size and sampling 

The total sample size calculated for the study with a population survey design in mind was n=105 subjects; the total population size in sub-urban Mumbai according to the 2011 census by the Government of Maharashtra was 93,56,962 [5]. The expected frequency of undernutrition according to the National Family Health Survey (NFHS)-5 (2019-21) was 36% with an acceptable margin of error of 6% [6]. Sample size calculation was done using CDC EpiInfo’s StatCalc software ver. 7.2.5.0, using the above-mentioned expected frequency and population size. 

Statistical analysis 

The data on categorical variables is shown as n (% of cases) and the data on continuous variables is presented as mean and SD or median and interquartile range (IQR). The inter-group statistical comparison of categorical variables is done using the chi-squared test. The inter-group statistical comparison of continuous variables is done using the independent samples t-test (for two groups). The underlying assumptions for normality and equality of variances were tested before subjecting the study variables to the independent samples t-test. Analysis of variance (ANOVA) test was applied for evaluating the association between the variables. 

In the entire study, p-values <0.05 are considered to be statistically significant. All the hypotheses were formulated using two-tailed alternatives against each null hypothesis (hypothesis of no difference). The entire data is statistically analyzed using JASP 0.16.2.0 for MS Windows.

## Results

A total of 126 children formed our study sample (Table 1). This included 53 boys (42.06%) and 73 girls (57.94%). For the majority (84.13%) of these children, the caregivers were mothers (n=106). The other caregivers responsible for the children included fathers (n=4; 3.18%), grandmothers (n=5; 3.97%), sisters (n=5; 3.97%), and aunts (n=6; 4.76%). 

**Table 1:** Study sample demographics SD: standard deviation

Parameter	Mean	SD
Age of the child	3.58 years	2.49 years
Age of primary caregiver	29.99 years	7.74 years
Weight of the child	12.07 kg	5.17 kg
Height/length of the child	86.75 cm	20.45 cm
Mid-arm circumference	14.69 cm	2.07 cm
Waist circumference	47.09 cm	9.38 cm

Nutrition status

The mid-arm circumference is a very useful age-independent parameter that can be used to assess pediatric nutritional status. Among the kids surveyed, 109 of them (86.50%) had a mid-arm circumference of more than 12.5 cm (normal), 11 (8.73%) were between 11.5 cm and 12.5 cm (moderate acute malnutrition), and five (4.77%) were less than 11.5 cm (severe acute malnutrition). BMI for age Z-score is another very useful marker that can be used in kids above two years of age. Among the 86 kids that belonged to this population in our study sample, 36 (44.19%) were found to be underweight (<5th percentile) while 14 (16.3%) were obese (>95th percentile) and four (4.65%) were overweight (85th-95th percentile). The remaining 30 (34.88%) had normal BMI. The accuracy of BMI perception by the caregivers was also noted in our study. While 39 caregivers (45.35%) accurately estimated the BMI category of their child, 25 caregivers (29.07%) overestimated their children’s BMI, labeling their "underweight" wards as "normal." Interestingly, 22 (25.58) caregivers underestimated the kids’ BMI categories. 

Educational status

Out of these 126 children, 70 (55.55%) children were found to be eligible to receive formal education while the remaining 56 (44.45%) were too young to meet the age eligibility criteria to be enrolled. Out of those who had commenced receiving formal education, only 39 (55.71%) were in an appropriate grade for their age while the other 31 (44.29%) were too old for their grade.

Diet patterns

Among the study sample, 22 children (17.46%) were administered a vegetarian diet, 11 (8.73%) were given a predominantly egg-based diet and the remaining 93 (73.81%) were partaking in a non-vegetarian diet. The mean expenditure on food as a proportion of the total household income was 36.40% (SD 15.0%) in the study sample. 

Milk was consumed routinely after food by 73 children (57.94%) while the other 53 kids (42.06%) consumed milk before food. Close to half the kids (n=51; 40.48%) consumed milk around breakfast, 44 (34.92%) consumed it around lunch while the remaining 31 (24.60%) consumed milk around dinner time. Since the study sample included children (age <5 years), three suitable parameters from the Children’s Eating Behavior Questionnaire were assessed [7]. These reflected food-approaching behaviors ("desire to drink" and "food responsiveness") as well as food-avoidant behaviors ("satiety responsiveness"). The correlation of these behaviors with the nutritional status (mid-arm circumference) as well as sleep quality score was not found to be statistically significant for our sample. 

Sleep patterns

No night-time awakenings were reported by 104 children (82.54%). On the single-item sleep quality scale, the sleep of 36 kids (28.58%) was reported by their caregivers as excellent, 81 kids (64.28%) as good, seven (5.55%) reported fair, and only two (1.59%) represented poor sleep quality. With respect to sleep duration, eight kids (6.34%) had a regular sleep duration of fewer than six hours, 90 kids (71.44%) had a sleep duration of six to nine hours, and 28 kids (22.22%) slept for more than nine hours every day. 

Medical history

Though data was collected during the COVID-19 pandemic, a high proportion of other symptoms were also seen among the children with 12 (9.52%) of them reporting abdominal bloating, 14 (11.11%) having abdominal pain, and 24 (19.05%) taking prescription drugs including antibiotics and antipyretics for respiratory illnesses in the past month. Hospitalization was needed in 12 (9.52%) children with seven (5.56%) having a history of surgery due to congenital causes like clubfoot and secondary to trauma injuries. 

The association between pediatric BMI and food expenditure as a proportion of total income was found to be positive but statistically insignificant (p-value=0.067). ANOVA test also showed a significant association between mid-arm circumference and the child receiving formal education (p-value=0.024). Other associations and correlations yielded non-significant results.

## Discussion

As a developing country, India faces immense pressure to undergo urbanization in a short period far exceeding the resources that can be provided leading to the growth of slums. The question arises then what is a slum? This can be answered through the definition provided by the UN-Habitat which has given certain indicators that if lacking in a household is defined as a slum. The indicators are lack of access to improved water sources or sanitation facilities, lack of sufficient living areas, lack of household durability, and lack of secure tenure [8]. Indian cities are the engines that propel the country ahead with Mumbai being dubbed the finance capital of India. Yet, slums due to their poor living conditions take a heavy toll on the population's social and physical health, thereby causing a downturn in the country's progress [9]. Hence assessment of malnutrition determinants remains an essential lynchpin in finding the efficacy of public health policies implemented by the country. Our study assesses the magnitude and determinants of malnutrition in an urban slum in India consisting of 126 study participants. The study participants were below the age of five with their main caregiver being the mother (84.13%). It is essential to note the education of the child as it serves as an indirect indicator of the families' socio-economic status. In our study, 44.29% of the study populace eligible for education were old for their grade. There can be two hypotheses for this finding: first, as slum dweller, they would find themselves caught in the cycle of poverty, and a positive correlation has been found between poverty and delayed entry into schools compared to their peers. The second reason could be the inter-relationship between nutrition and cognitive development. A significant period for cognitive development is the ages of zero to two years, and the brain is sensitive to any nutritional insults at that time [10]. A study in Africa found that after controlling of all variables, the deficit remained stable across ages [11]. Therefore our study participants, out of which a significant number of them are suffering from malnutrition could find their cognitive development hampered. 

The release of the NFHS 5 for the year 2019-2021, which was conducted by the Ministry of Health and Family Welfare, heralded a stunning reduction in the incidence of malnutrition. The prevalence of underweight has reduced to 32.1%, as opposed to 35.8%, which was found in the NFHS-4 [12]. Our study finds the prevalence of underweight to be 44.19%. This prevalence is similar to those found in previous studies. Murarkar S et al. find the prevalence of underweight in urban slums of Maharashtra to be 35.4% [13]. Purohit L et al. find in their community-based study in a city in Maharashtra that the total magnitude of underweight is 38.15% [14]. In a study conducted in West Bengal, the underweight prevalence was 41% [15]. 

Urbanization has also been implicated in both undernutrition as well as overnutrition. Overnutrition leads to obesity, which can cause deleterious health effects in children. Our study is the first study to find the prevalence of overweight and obese children in an urban slum in Mumbai. The prevalence is 4.65% and 16.3%, respectively. This double burden of malnutrition can be attributed to the consumption of heavy energy-dense food [16]. Being obese as a child has a positive correlation to being obese as an adult, which can then predispose the individual to a variety of lifestyle diseases [17]. 

In the age group of 0-59 months, the incidence of stunting and wasting is determined to be 62.5% and 26.6%, respectively. Previous studies found the prevalence to be 40.46% and 51% for stunting and wasting, respectively, in Maharashtra [14]. Stunting is an important anthropometric parameter as it is an indicator of chronic undernutrition while wasting implies a child who has recently suffered from food deficit or illness. Our study also noted that poor anthropometric growth scores were found in males than females, which reinforces the results found in earlier studies conducted [2,18,19]. We also noted that despite the children suffering from moderate or severe malnutrition, there were a significant number of mothers who believed that their children's current anthropometric parameters were normal. This poor understanding of malnutrition can lead to undernourished children receiving delayed medical interventions.

Malnutrition has always been linked to infection in a self-reinforcing cycle. Pooja G et al. reported that children who had suffered from respiratory pathology, viral illness, diarrhea, and vomiting in the past three months before the initiation of data collection were at a higher risk of being wasted, stunted, or malnourished [20]. Similar results were also obtained in studies conducted by Sarkar et al. and Singh et al. [21,22]. Our study notes that 19.05% were taking prescription drugs including antibiotics and antipyretics for respiratory pathologies. Around 20.63% of children also complained of GI symptoms. There were also 9.52% of the children hospitalized with a major reason being to undergo surgery to correct their congenital disease.

In India, special nutritional programs have been curated for children for the prevention of malnutrition such as ICDS scheme, Mid-Day Meal (MDM) Programs, Special Nutrition Programs (SNP), and Balwadi Nutrition Programs (BNP) [23]. Out of these, the MDM program is availed by children going to school. Hence children's education becomes an important parameter in identifying interventions to ameliorate malnutrition. Our study finds that 44.45% of the children interviewed were not eligible for schooling and consequently did not receive the benefits of the MDM scheme. Our study is unable to find data on whether the child or the caregiver attended a Balwadi or an Anganwadi; hence we cannot comment on the utilization of the other programs.

No statistically significant association was found between the participants' food-approaching or food-avoidant behaviors and nutritional status. It is important to find whether such a correlation exists as child eating habits are an important determinant in understanding the etiology of malnutrition. Picky or fussy eaters are prone to suffering from micronutrient deficiencies [24]. These micronutrient deficiencies can predispose the child to infection, thereby initiating the vicious malnutrition infection cycle. On the other spectrum children with decreased satiety are prone to overeating and can become overweight/obese down the line [25]. 

Diet and sleep have a complex inter-relationship through their impact on regulatory hormonal pathways. A poor diet predisposes to an erratic sleep cycle while diminished sleep can contribute to dysregulated eating patterns [26]. Dysregulated eating patterns can contribute to obesity down the line and the adverse health outcomes that are associated with it. These habits are found to be established in early childhood and continue as the child ages. In our study, despite the high prevalence of malnutrition, the majority of our study participants slept for six to nine hours with their caregivers rating their sleep as either excellent or good with no night-time awakening. Their diet was predominantly non-vegetarian, and milk was consumed routinely after food around breakfast. Our study, thereby, finds no relation between diet and sleep. 

Our study had few limitations. The study has a small sample size of only 126 participants (although statistically sufficient) and over- and under-reporting of data could have occurred by the caregivers. Therefore, we suggest extensive large and multicentric studies in the slum areas of India to assess the masked iceberg of malnutrition in slums of metropolitan cities.

## Conclusions

The current schemes by the Indian government as well as previous studies on the topic target malnutrition on a national scale, and targeted focus on high endemic areas of malnutrition is missing. Our study in contrast takes a narrowed region-wise focus on malnutrition in India. Our study reports a substantial burden of malnutrition among children residing in the slums of Dharavi. Hence, rigorous strengthening and conceptualization of on-ground nutritional programs targeted toward slum children should be done by Indian healthcare policymakers.
